# Esterification of geraniol as a strategy for increasing product titre and specificity in engineered *Escherichia coli*

**DOI:** 10.1186/s12934-019-1130-0

**Published:** 2019-06-08

**Authors:** Micaela G. Chacón, Alice Marriott, Emanuele G. Kendrick, Matthew Q. Styles, David J. Leak

**Affiliations:** Department of Biology and Biochemistry, University of Bath, Bath, England BA2 7AY UK

**Keywords:** *Escherichia coli*, Geraniol, Geranyl acetate, Alcohol acyltransferase, Microbial production

## Abstract

**Background:**

Geraniol, an acyclic monoterpene alcohol, is found as a primary constituent in the essential oils of plants such as geranium, lemongrass and rose. The floral-like scent of geraniol has made it a popular constituent of flavour and fragrance products. Over recent decades biotechnology has made significant progress towards the development of industrial platforms for the production of commercially valuable monoterpenoids, such as geraniol, through expression of recombinant terpene biosynthetic pathways in microbial hosts. Titres, however, have been hindered due to the inherent toxicity of these compounds—which are often utilised for anti-microbial and anti-fungal functions in their host plant.

**Results:**

In this study we modified an *Escherichia coli* strain, engineered to express a heterologous mevalonate pathway, by replacement of the terpene synthase with a geraniol synthase from *Ocimum basilicum* for the production of geraniol, and co-expressed an alcohol acyltransferase (AAT) from *Rosa hybrida* for the specific acetylation of geraniol. The low water solubility of geranyl acetate facilitated its partition into the organic phase of a two-phase system, relieving the cellular toxicity attributed to the build-up of geraniol in the aqueous phase. In a partially optimised system this strain produced 4.8 g/L geranyl acetate (based on the aqueous volume) which, on a molar equivalent basis, represents the highest monoterpene titre achieved from microbial culture to date. It was also found that esterification of geraniol prevented bioconversion into other monoterpenoids, leading to a significant improvement in product specificity, with geranyl acetate being the sole product observed.

**Conclusion:**

In this study we have shown that it is possible to both overcome the toxicity limit impeding the production of the monoterpene alcohol geraniol and mitigate product loss in culture through endogenous metabolism by using an in vivo esterification strategy. This strategy has resulted in the highest geraniol (equivalent) titres achieved from a microbial host, and presents esterification as a viable approach to increasing the titres obtained in microbial monoterpenoid production.

**Electronic supplementary material:**

The online version of this article (10.1186/s12934-019-1130-0) contains supplementary material, which is available to authorized users.

## Introduction

Geraniol (trans-3,7-dimethyl-2,6-octadien-1-ol) is an acyclic monoterpene alcohol found in the essential oils of plants such as lemongrass, rose, and geranium [1]. Due to its distinctly sweet and floral aroma it has become a fundamental commercial fragrance compound, with an extensive survey confirming its presence in 40% of deodorants, 58% of perfumes and 26% of cosmetic creams [2]. As such it represents a key product within the global flavour and fragrance industry, which was valued at 18.6 billion (USD) in 2015 [3]. More recently, additional commercial applications for geraniol have been proposed, including use as a pharmaceutical agent for anti-cancer, anti-inflammatory, and pain relief purposes; and as a promising gasoline alternative [4–8]. Currently, the predominant strategies for obtaining terpenes such as geraniol are extraction from plant material and chemical synthesis; both of which are costly and inefficient [9, 10]. As such, there has been growing interest in developing more sustainable and economical biotechnological methods for terpene production [11].

Terpenoids are also attractive targets from the perspective of microbial engineering, as all terpenoids are produced from a single universal precursor, isopentenyl pyrophosphate (IPP) [12]. A microbial strain engineered to produce high levels of IPP can form the chassis to drive the biosynthesis of numerous terpenoids with the heterologous production of just two additional proteins; the correct terpene synthase, and a geranyl pyrophosphate synthase (GPPS) for C10 monoterpenes or a farnesyl pyrophosphate synthase (FPPS) for C15 sesquiterpenes. This approach has been widely used for microbial sesquiterpene production, with several commercial examples, including the production of farnesene, valencene, and *trans*-nerolidol [13, 14]. However, there are no examples yet of recombinant microbes capable of producing commercially viable titres of monoterpene products, with maximum titres in almost all cases being in the 100 mg/L range [15], compared to ~ 100 g/L achieved for some sesquiterpenes [16, 17].

The obstacles impeding high geraniol production include (i) its high microbial toxicity and (ii) product loss due to bioconversion by endogenous *E. coli* enzymes to other monoterpenoids, which are themselves toxic to various degrees [18–23]. Geraniol toxicity has been attributed to its amphiphilic nature, allowing it to interact with cell membranes, impacting their integrity and permeability, as well as with intracellular components [18, 20]. Shah et al. [24] demonstrated that geraniol exposure in *E. coli* causes DNA damage, confirming its multifaceted mode of toxicity. In addition to toxicity, several groups have reported metabolism of endogenously produced geraniol in *E coli* cultures to other geranoids (geranial, nerol, neral) and monoterpenoids such as citronellol and linalool [21, 22]. This has been attributed to the presence of promiscuous *E. coli* enzymes such as yjgB, a geraniol dehydrogenase that converts geraniol to geranial [22]. Both toxicity and further metabolism need to be addressed in order to achieve industrially viable geraniol titres. To do this, we envisaged that the deliberate conversion of geraniol to a more hydrophobic ester could both improve production by facilitating its extraction into the organic layer of a two-phase culture and protect the alcohol group from further metabolism. Fortuitous partial acetylation of monoterpenoids via the promiscuous activity of a chloramphenicol acetyltransferase (CAT) has previously been reported for geraniol and perillyl alcohol [8, 25, 26].

**Fig. 1:**
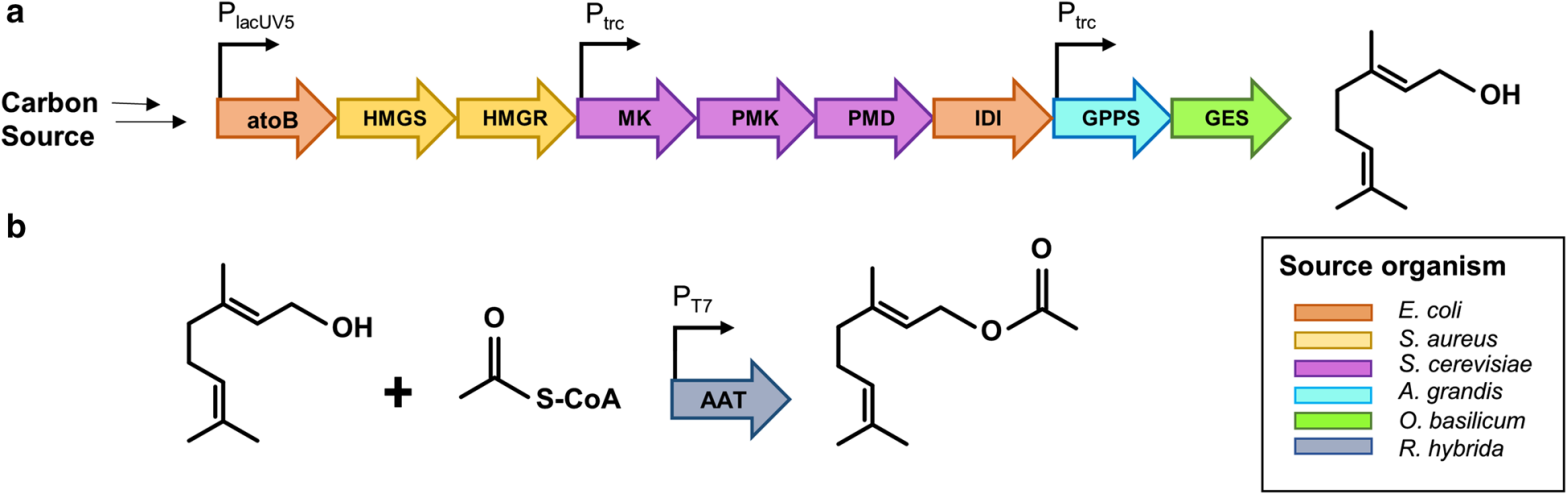
Diagram of the two *E. coli* expression constructs used in this study harbouring **a** the heterologous mevalonate pathway (MEV) leading towards the production of geraniol as expressed by pGER, and **b** an alcohol acyltransferase (AAT) capable of esterifying geraniol and acetyl-CoA to produce geranyl acetate as expressed by pET28a::*Rh*AAT. Co-transformation of *E. coli* with the two plasmids, results in the production of geraniol and in vivo conversion to a geranyl acetate ester in high titres. The origin of all enzymes expressed on each plasmid is colour coded according to species of origin (inset)

In this paper we describe the engineering of *E. coli* to produce geranyl acetate from glucose through heterologous expression of the mevalonate pathway, a geraniol synthase (GES) from *Ocimum basilicum*, and an alcohol acyltransferase (AAT) from *Rosa hybrida* with distinct substrate specificity for geraniol (Fig. 1). By engineering a system that converts endogenously made geraniol immediately into geranyl acetate we have been able to significantly improve titres. Further, this acetylation appears to halt endogenous conversion of geraniol to other monoterpenoids, resulting in a single end-product.

## Materials and methods

All media components, solvents and chemicals were purchased from Sigma-Aldrich (Dorset, UK) or Fisher Scientific (Loughborough, UK) unless otherwise stated. *E. coli* strains BIO*Blue* (Bioline; London, UK) and C43 (DE3) (Lucigen; Middleton, WI) were used for plasmid construction and expression, respectively. Gene sequence optimization and synthesis was performed by GeneArt (Thermo Fisher Scientific; Waltham, MA). Plasmid pJBEI-6410 was purchased from Addgene as plasmid # 47049 (a gift from Taek Soon Lee).

### Plasmids, bacterial strains, and growth conditions

All plasmids and strains used in this study are listed in Table 1. *E. coli* strain C43 (DE3) was chosen as the expression host as it demonstrates high tolerance to toxicity associated with recombinant protein over-expression in a bacteriophage T7 RNA polymerase system [27]. Lysogeny Broth (LB, 10 g/L tryptone, 10 g/L NaCl, and 5 g/L yeast extract) was used for pre-culturing.

**Table 1:** Description of *E. coli* plasmids and strains used in this study

Name	Description	References
Plasmids		
pET28a::*Rh*AAT	pColE1, Kan^R^, T7-*Rh*AAT	This study
pJBEI-6410	p15a, Amp^R^, PlacUV5-*Ec*AtoB-*Sa*HMGS-*Sa*HMGR-T1^a^, Ptrc-*Sc*MK-*Sc*PMK-*Sc*PMD-*Ec*idi-T1002^b^, Ptrc-*Ag*trGPPS_-_*Ms*LS	Alonso-Gutierrez et al. [25]
pGER	p15a, Amp^R^, PlacUV5-*Ec*AtoB_-_*Sa*HMGS-*Sa*HMGR-T1, Ptrc-*Sc*MK-*Sc*PMK_-_*Sc*PMD-*Ec*idi-T1002, Ptrc-*Ag*trGPPS_-_*Ob*GS	This study
Strains		
BIO*Blue*	recA1, endA1 gyrA96 thi-1 hsdR17(r_k_-, m_k_ +) supE44 relA1 lac [F’ proAB lacI^q^ZΔM15 Tn10(Tet^r^)]	Bioline
C43 (DE3)	F-ompT hsdSB (rB- mB-) gal dcm (DE3)	Lucigen
DLG	C43 (DE3) harbouring pGER	This study
DLGA	C43 (DE3) harbouring pET28a::*Rh*AAT	This study
DLGA1	C43 (DE3) harbouring pGER and pET28a::*Rh*AAT	This study

In the plasmid description, subscript letters refer to the source of the gene as follows: *Rh* = *Rosa hybrid; Ec* = *Escherichia coli; Sa* = *Staphylococcus aureus*; *Sc* = *Saccharomyces cerevisiae*; *Ab* = *Abies grandis*; *Ms* = *Mentha spicate*; *Ob* = *Ocimum basilicum*

^a^T1: both the rrnB T1 terminator and T7Te terminator

^b^T002: terminator

For shake-flask production of geraniol or geranyl acetate, recombinant strains were cultured in Terrific Broth (TB, 12 g/L tryptone, 24 g/L yeast extract, 4 mL glycerol, 0.17 M KH_2_PO_4_, and 0.72 M K_2_HPO_4_) containing 20 g/L glucose. For fed-batch production of geraniol or geranyl acetate in a bioreactor, recombinant strains were cultured in either a modified TB (MTB, 20 g/L glucose, 12 g/L tryptone, 24 g/L yeast extract, 4 mL glycerol, 5 g/L NaCl), or a semi-defined fermentation (FM) media (20 g/L glucose, 9.8 g/L K_2_HPO_4_, 5 g/L yeast extract, 0.3 g/L ferric ammonium citrate, 2.1 g/L citric acid monohydrate, 0.06 g/L MgSO_4_ and 1 mL of trace element solution which includes 0.37 g/L (NH_4_)_6_Mo_7_O_24_·4H_2_O, 0.29 g/L ZnSO_4_·7H_2_O, 2.47 g/L H_3_BO_4_, 0.25 g/L CuSO_4_·5H_2_O and 1.58 g/L MnCl_2_·4H_2_O). Ampicillin (Amp, 100 µg/mL) or kanamycin (Kan, 50 µg/mL) were added to the culture medium depending on the selectable marker gene on each plasmid.

### Vector construction

All recombinant plasmids were sequenced for verification by GATC Biotech (Konstanz, Germany). The codon-optimized gene sequence of AAT from *R. hybrida* (*Rh*AAT) (GenBank No. AY850287.1) was cloned between restriction sites *Bam*HI and *Xho*I in the IPTG-inducible *E. coli* expression vector pET28(a) to produce pET28a::*Rh*AAT. To create pGER, the limonene synthase gene (LS) in plasmid pJBEI-6410 (Alonso-Gutierrez et al. [25]) was replaced with the full-length codon optimized sequence of geraniol synthase (GES) from *O. basilicum* (GenBank No. AY362553.1) (Additional file 1: Fig. S1) using Gibson assembly. This was done by digesting the pJBEI-6410 plasmid at restriction sites *Kpn*I and *Bam*HI—the former site located 185 bp into the upstream Geranyl Pyrophosphate Synthase (GPPS) gene, and the latter located directly downstream of the LS gene. A DNA fragment composed of the remaining 709 bp of the upstream GPPS gene followed by a *Sal*I restriction site and the GES gene was flanked on either end with Gibson overhangs complementary to the vector sequence (forward primer: GTTCTGTGTATTGCCGCAT reverse primer: TTTATTTGATGCCTGGAGATCCTTACTCGAGTTTGGATCCTTACTGGGTAAAAAACAGGGC) and inserted into pJBEI-6410 to reconstitute the GPPS gene and replace the limonene synthase with the geraniol synthase gene. The insertion of a *Sal*I restriction site was done to facilitate any future exchange of the terpene synthase.

### Geraniol toxicity assays

Toxicity assays were carried out as described by Dunlop et al. [28]. C43 (DE3) was inoculated into 5 mL of LB and grown overnight at 37 °C. Cultures were then inoculated in triplicate to a starting OD_600_ of 0.05 in a 24-well plate in 1 mL of TB + 20 g/L glucose medium ± varying concentrations of geraniol or geranyl acetate. A growth time course was carried out at 37 °C in a plate reader (BioTek Synergy 4, USA) operating at a shaking intensity of 4, by measuring the OD_600_ every 5 min for 8.5 h.

### Shake-flask geraniol and geranyl acetate production

Strains DLG and DLGA1 were inoculated into 5 mL of LB medium supplemented with the relevant antibiotics and grown overnight at 37 °C. Cultures were then inoculated to 1% (v/v) in 25 mL of TB + 20 g/L glucose medium in a 250 mL baffled flask and grown at 37 °C to an OD_600_ of 1. Cultures of the DLG strain were then induced with 50 µM IPTG, a 10% (v/v) dodecane top layer was added and flasks incubated on a rotary shaker (250 rpm) at 30 °C. After 24 h the OD_600_ in the aqueous phase was measured and a sample of the dodecane layer was taken and diluted into ethyl acetate for analysis by gas-chromatography mass spectometry (GC–MS).

Cultures of the DLGA1 strain were induced with 100 µM IPTG and, where indicated, supplemented with either 5, 10 or 20 mM acetic acid before addition of a 10% (v/v) dodecane top layer. Cultures were then incubated and analysed as described above.

### Production of geraniol and geranyl acetate in bioreactors

DLG and DLGA1 grown overnight at 37 °C in 100 mL LB were used to inoculate a 1.5 L (working volume) bioreactor (BIOSTAT B, Sartorius, Germany) containing 1.2 L MTB supplemented with the relevant antibiotics maintained at pH 6.8 (by automatic addition of 5 M KOH) and 30 °C. Antifoam 204 was added on demand and dissolved oxygen was maintained at 20% saturation through combined control of air flow and stirrer speed (maintained between 0.5–1 L/min and 300–600 rpm, respectively). Intermittent feeding of an MTB + 65 g/L glucose solution was initiated to maintain culture glucose concentration between 5 and 10 g/L. DLG was induced with 50 µM IPTG when the OD_600_ reached approximately 20, and a 10% dodecane top layer was then added. For DLGA1 125 µM IPTG was used and 20 mM acetic acid was added together with the 10% dodecane. Fermentation samples were collected periodically, and OD_600_ was determined prior to brief centrifugation to separate the organic and aqueous phase. The organic layer was removed for GC–MS analysis while the aqueous layer was removed for IC analysis.

### Aqueous and organic partition of geraniol and geranyl acetate

10 mL of TB + 2% glucose media was supplemented with either 100 mg/L of geraniol or geranyl acetate followed by the addition of a 10% dodecane top layer before being incubated at 30 °C and 250 rpm for 6 h. After incubation, a sample of the dodecane was taken directly for analysis by GC–MS, while the aqueous layer was further extracted with hexane before analysis.

### Quantification of metabolites

#### Product characterization by GC–MS

Terpene and ester products were quantified using a model 7890B gas chromatograph linked to a 5977A mass spectrometer (Agilent technologies, Stockport, UK). Samples were separated on a DB-FFAP 30 m × 20 µm, d_f_ 0.25 µm capillary column under the following conditions: 1 µL sample volume, column temperature 40 °C for 1 min, followed by a 20 °C/min gradient to 250 °C which was held for 8 min. Terpene and ester products typically eluted between 7 and 11 min and were identified by both MS (comparison to the NIST Mass Spectral Library) and retention time compared to authentic standards. Product concentration was quantified using FID calibration curves of authentic standards. Samples for the calibration curves were constructed by extracting 5 mL of TB + 2% glucose media containing a discrete amount of terpene or ester product into 500 µL of dodecane, to take account of extraction efficiency.

#### Glucose quantification by Ion Chromatography (IC)

Glucose concentrations in filtered culture broth were monitored by ion chromatography (Dionex 5000 +) using a 4 × 250 mm analytical CarboPac PA1 column (Thermo Fisher Scientific, USA). Analytes separated isocratically at 30ºC using 50 mM NaOH as eluent at a flow rate of 1.0 mL/min.

## Results and discussion

### Geraniol and geranyl acetate toxicity

**Fig. 2:**
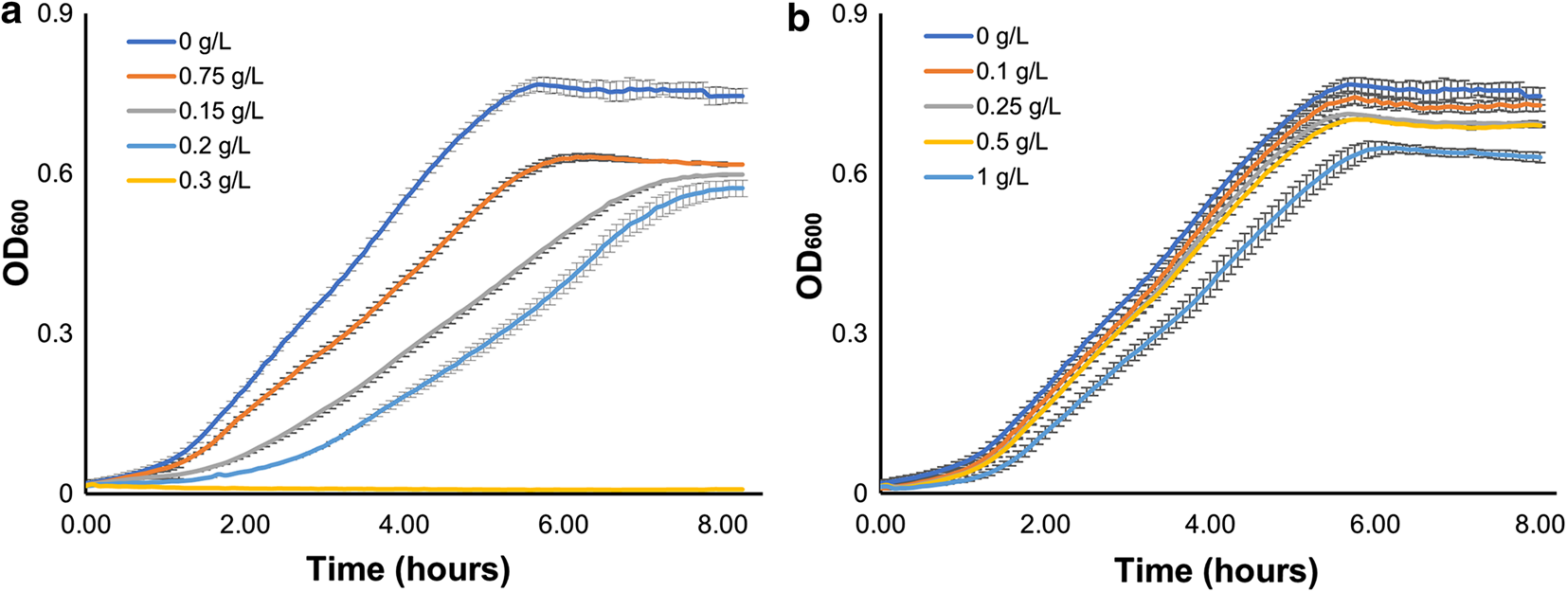
Growth of wild type *E. coli* strain C43 (DE3) in the presence of **a** 0, 0.075, 0.15, 0.2, or 0.3 g/L geraniol or **b** 0, 0.1, 0.25, 0.5, or 1 g/L geranyl acetate. Data are the mean ± standard deviation of three biological replicates

The antimicrobial action of geraniol has previously been demonstrated against a number of bacterial and fungal species, with Dunlop et al. [28] demonstrating that 0.05% (v/v) geraniol (444 mg/L) completely inhibited growth of *E. coli*. As a result, recorded titres for microbial geraniol production are low—even with the use of an extractive organic phase in culture, as the amphiphilic nature of geraniol prohibits complete removal from the aqueous phase. Table 2 shows the aqueous phase partition of 100 mg/L geraniol in an equilibrated biphasic system using a 10% dodecane top-layer. Here, 66% of geraniol was extracted, leaving 34% (34 mg/L) in the aqueous phase where it has the potential to exert toxic stress on the microbial cells. Toxicity tests show that *E. coli* strain C43 (DE3) had a reduced growth rate in the presence of 75 mg/L geraniol in the aqueous phase, while 300 mg/L inhibited growth entirely (Fig. 2). Together, these data are consistent with the highest microbially produced titres of geraniol reported thus far in the literature [15, 22, 26].

**Table 2:** Partition of geraniol and geranyl acetate into the aqueous and organic layers of a biphasic system

	% Aqueous	% Top layer	Partition [Product_Toplayer_]/[Product_aq_]
Geraniol^a^	34 ± 3	66 ± 7	19.4
Geranyl acetate^a^	4 ± 0.6	96 ± 2	240

^a^TB + 2% glucose with a 10% dodecane top layer was supplemented with 100 mg/L geraniol or geranyl acetate and incubated for 3 h before analysis. Data are the mean ± standard deviation from three biological replicates

Geranyl acetate, in contrast, exerts less toxic stress on *E. coli*, with a concentration of 1 g/L in the aqueous phase beginning to have an impact on growth (Fig. 2). Dunlop et al. [28] found that the addition of 4.6 g/L of geranyl acetate to an aqueous culture resulted in a 40% reduction final OD_600_, but that further addition of geranyl acetate, up to a nominal 46 g/L, caused no additional reduction in OD_600_, possibly due to its low aqueous solubility, above which it will form a second phase. It is unclear to what extent geranyl acetate itself is toxic, as it is found to be hydrolysed by endogenous *E. coli* enzymes to geraniol and other products when supplemented in the culture (Additional file 1: Fig. S5). As geranyl acetate is less water-soluble than geraniol it extracts more efficiently into an organic phase. Table 2 demonstrates that a nominal 96% of 100 mg/L geranyl acetate is extracted into dodecane in an equilibrated biphasic system. This suggests that the simple conversion of geraniol into its acetate ester could not only improve in situ product removal into an organic phase but also possibly drive higher productivity by limiting exposure of cells to toxic levels of geraniol.

### Geraniol and geranyl acetate production in *E. coli*

**Fig. 3:**
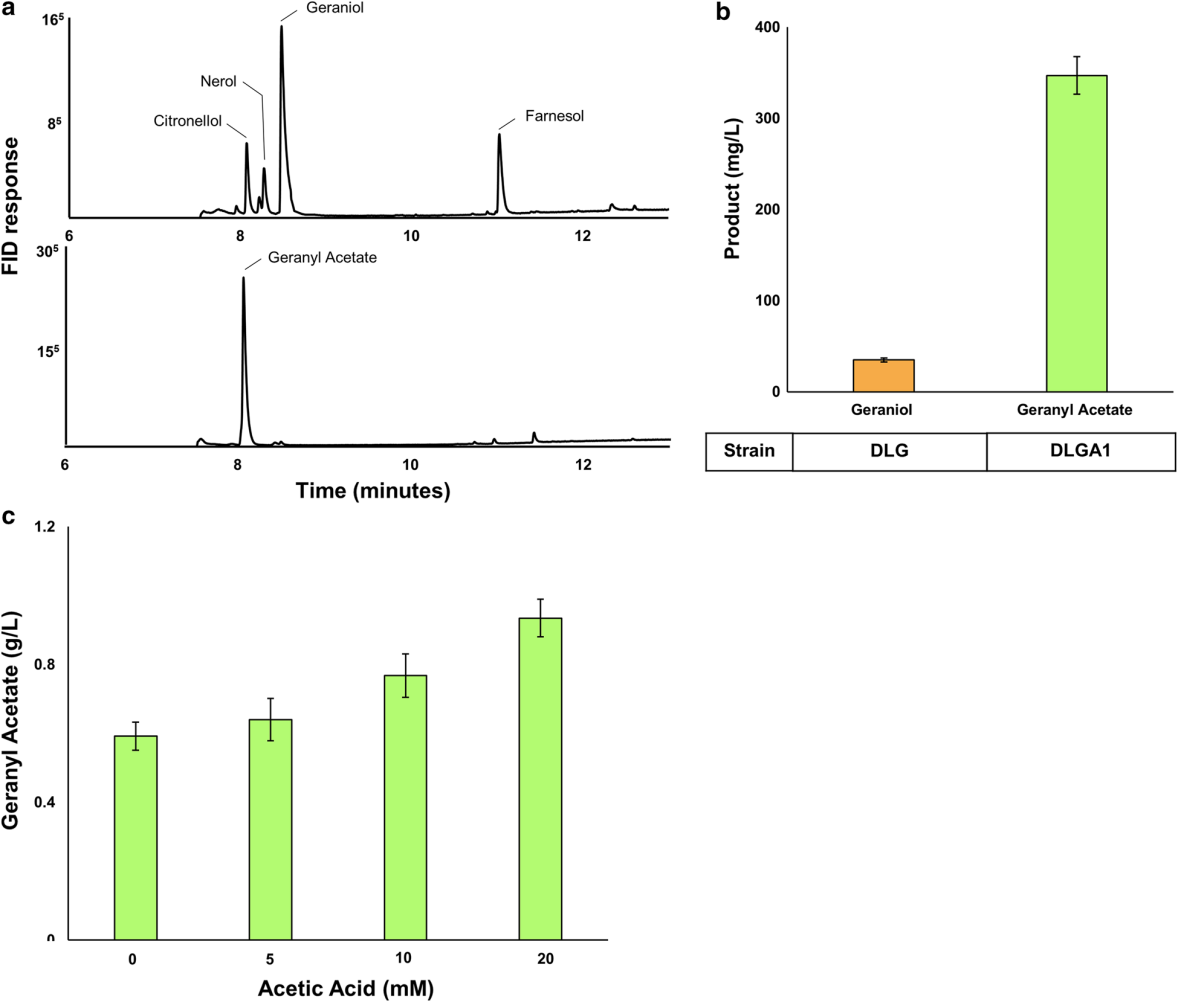
**a** Gas chromatographs of the dodecane phase of two-phase cultures of *E. coli* strains DLG (top panel) producing several terpenols, and DLGA1 (bottom panel) producing only geranyl acetate. **b** The total production (mg/L) of either geraniol or geranyl acetate produced by strain DLG and DLGA1, respectively in 250 mL non-baffled flasks. **c** Geranyl acetate production by *E. coli* strain DLGA1 after 24 h when supplemented with 5 mM, 10 mM or 20 mM acetic acid in 250 mL baffled flasks. Products were analysed after 24 h of growth at 30 °C. Chromatograph peaks for citronellol, nerol, geraniol, farnesol, and geranyl acetate are indicated. Data are the mean ± standard deviation from three biological replicates

The previously described plasmid, pJBEI6410 [25], encoding an optimized MEV pathway terminated by a limonene synthase, was modified for the production of geraniol (Fig. 1). This was done by replacing the limonene synthase gene (LS) with a geraniol synthase gene (GES) from *O. basilicum*—resulting in the plasmid pGER. The full-length coding sequence of the GES gene was codon-optimized for expression in *E. coli*, including its N-terminal plastidial targeting sequence which was not removed as it had previously been demonstrated not to interfere with the expression or activity of the recombinant enzyme [8, 29]. *E. coli* C43 (DE3) was transformed with plasmid pGER to produce strain DLG (Table 1) which, in initial experiments, produced the equivalent of 35 mg/L (aqueous volume) of geraniol after 24 h when expressed in *E. coli* in a two-phase culture (Fig. 3b).

In addition to producing geraniol, this strain produced significant amounts of the monoterpenoids, nerol and citronellol, as well as the sesquiterpenoid farnesol (Fig. 3a). Previous work has determined that the GES enzyme from *O. basilicum* has strict specificity for geraniol production from geranyl pyrophosphate [29], suggesting that the production of additional monoterpene products is the result of further metabolism of aqueous phase geraniol by endogenous *E. coli* enzymes. Fisher et al. [21] have previously demonstrated that the terpene profile obtained from GES expression is heavily dependent on the heterologous host in which it is being expressed. The production of farnesol suggests that the activity of GES is insufficient to trap all the geranyl pyrophosphate produced and that some farnesyl pyrophosphate was also being made. As further metabolism of geraniol is undesirable, several groups have worked toward minimizing it through genetic manipulation of the heterologous host [22]. However, the in situ esterification of geraniol would not only produce a more hydrophobic product but also potentially reduce the propensity for further metabolism.

In order to convert geraniol to geranyl acetate, an alcohol acyltransferase (AAT) from Rose (*Rosa hybrida*) with reported activity for catalysing the esterification of geraniol by acetyl-CoA [30] (Fig. 1) was expressed, initially in *E. coli* strain C43(DE3). The coding sequence of this AAT (*Rh*AAT) was optimized for expression in *E. coli* and cloned into pET28a to drive expression under the strong T7 promoter (Table 1). High AAT expression was desirable for this system as the efficient acetylation of geraniol is required to prevent this intermediate building up to toxic concentrations. *E. coli* C43(DE3) harbouring the pET28a::*Rh*AAT plasmid was named strain DLGA. Cultures of strain DLGA supplemented with 0.5 mM geraniol and 10 mM acetic acid produced geranyl acetate almost exclusively after an 18 h incubation (Additional file 1: Fig. S2; Additional file 1: Methods), with only trace amounts of citronellyl-, neryl- and linalyl acetate detectable by GC/MS (data not shown). As a control experiment, the *E. coli* strain C43 (DE3) without the pGER or pET28a::*Rh*AAT plasmids produced no geraniol or geranyl acetate (data not shown).

Co-transformation of *E. coli* with plasmids pGER and pET28a::*Rh*AAT generated strain DLGA1 (Table 1) with the metabolic potential to produce geranyl acetate entirely from acetyl-CoA. This strain was found to produce high titres of exclusively geranyl acetate (375 mg/L based on the aqueous volume) from glucose after 24 h in a two-phase culture (Fig. 3b). This is the molar equivalent of 1.9 mM geraniol, more than eightfold higher than the concentration produced by strain DLG. As the concentration of geraniol produced in 24 h by DLG was below that where toxic effects had been observed in initial studies and there was no evidence of cellular toxicity in either of these production studies (both DLG and DLGA1 were grown under the same culturing conditions and had no significant difference in final cell densities) this higher level of geraniol production was unexpected. Even taking into account the further metabolism of geraniol to nerol and citronellol there was still a fivefold increase in geraniol production when incorporated into geranyl acetate suggesting that, by removing geraniol as its ester a previously undetected restriction on flux to geraniol had been relieved. As a possible explanation, aqueous phase geraniol accumulation in strain DLG may have given rise to end-product feedback inhibition, limiting further geraniol production but not affecting growth. This was less likely to happen in DLGA1 because the geraniol was being sequestered as the acetate. In order to assess the potential for product inhibition of the GES enzyme, its in vitro activity was assayed in the presence of increasing concentrations of the product, geraniol. However, even at the highest geraniol concentrations tested, there was no significant impact on GES activity (Additional file 1: Table S1). Therefore, if the limit on geraniol production is the result of feedback inhibition, it must be occurring on a different element of the MEV pathway and not the terminal terpene synthase. Regardless of the mechanism, this appears to be another factor that can be overcome by conversion to an ester.

In addition to the improved product titres observed in strain DLGA1, there was also a marked improvement in product specificity—with geranyl acetate being the sole end-product (Fig. 3a). This suggests that the bioconversion of geraniol to nerol and citronellol requires the free alcohol and, that once esterified, further metabolism is either negligible or limited by the greater hydrophobicity of the geranyl acetate molecule, which partitions more effectively into the organic phase, sequestering it from endogenous enzymes (Fig. 3a, Additional file 1: Fig. S3). Further, the absence of any geraniol, nerol, or citronellol in cultures of strain DLGA1 indicate that the *Rh*AAT has high activity for the acetylation of geraniol—making this an effective targeted strategy. Overall, it was found that esterification of geraniol not only resulted in significantly improved final monoterpenoid titres, but also in improved final product specificity. The latter is an attractive feature as multiple end-products are a major problem hindering industrial microbial terpene production generally, as separation of similar terpenoid products is expensive.

### Acetic acid feeding to improve geranyl acetate production

To further improve geranyl acetate titres, potential pathway bottlenecks were considered. While the MEV pathway used here had been previously optimized to balance gene expression and flux of intermediates [25], accommodations for the increased consumption of acetyl-CoA had not been taken into consideration. Acetyl-CoA is a node from which much cellular metabolism branches. The introduction of a heterologous pathway that requires seven acetyl-CoA molecules for each geranyl acetate molecule imposes a significant demand on flux through this node. Previously it has been shown that genetic manipulations aimed at increasing acetyl-CoA precursor availability in *E. coli* and *S. cerevisiae* have resulted in improved product titres of the terpenes lycopene and amorphadiene, respectively [31, 32].

As an alternative approach, we investigated the viability of media supplementation with acetic acid as a means to increase the abundance of available acetyl-CoA. When grown in the presence of fermentable sugars, *E. coli* converts acetyl-CoA to acetate through the action of the reversible PTA-ACK pathway (using phosphotransacetylase/acetate kinase), with the acetate produced being subsequently excreted [33]. It has been found, however, that the direction of the PTA-ACK pathway is dependent upon the extracellular concentration of acetate, with acetate being assimilated by *E. coli* when present in an external concentration above a threshold value of 8 mM, even in the presence of excess glucose [34]. This suggests that the exogenous addition of acetic acid in *E. coli* cultures grown on glucose may be used as a strategy to increase the pool of intracellular acetyl-CoA as the driving force of the PTA-ACK pathway is pushed towards its synthesis, resulting in acetyl-CoA accumulation both from the consumption of glucose and acetate simultaneously [34–37]. Strain DLGA1 was cultured in media supplemented with either no acetic acid, 5 mM, 10 mM, or 20 mM acetic acid, which was added immediately post induction. After 24 h, cultures fed no acetic acid had produced 585 mg/L (based on aqueous volume) of geranyl acetate while the cultures supplemented with 20 mM acetic acid produced 940 mg/L; a 60% increase in product titre (Fig. 3c). This clearly demonstrated that the production of geranyl acetate in these strains was limited by the availability of acetyl-CoA, either as a precursor for geraniol biosynthesis or for acetylation of geraniol, and that further increases in flux to geranyl acetate by increasing acetyl-CoA production should be possible. Although high concentrations of acetic acid are detrimental to cell growth as it can uncouple the transmembrane pH gradient and inhibit methionine biosynthesis [33, 38], media supplementation with up to 20 mM acetic acid (1.2 g/L) had no significant impact on final culture OD_600_, most likely due to the buffering capacity of TB media (Fig. 3c).

Further improvement on this acetic acid supplementation strategy may be achieved by de-regulating expression of an acetyl-CoA synthase (ACS) enzyme—which catalyses the direct condensation of acetate and coenzyme A (CoA). Transcription of the endogenous *E. coli acs* is repressed in the presence of glucose [36]. Yang et al. [37] have recently shown that dual expression of an ACS from *Acetobacter pasteurianus* and an acetoacetyl CoA synthase (AACS) from *Streptomyces sp. strain CL190* in an *E. coli* strain engineered for β-caryophyllene production results in improved product titres when acetic acid was used as the sole carbon source.

### Geraniol and geranyl acetate formation in fed-batch culture

**Fig. 4:**
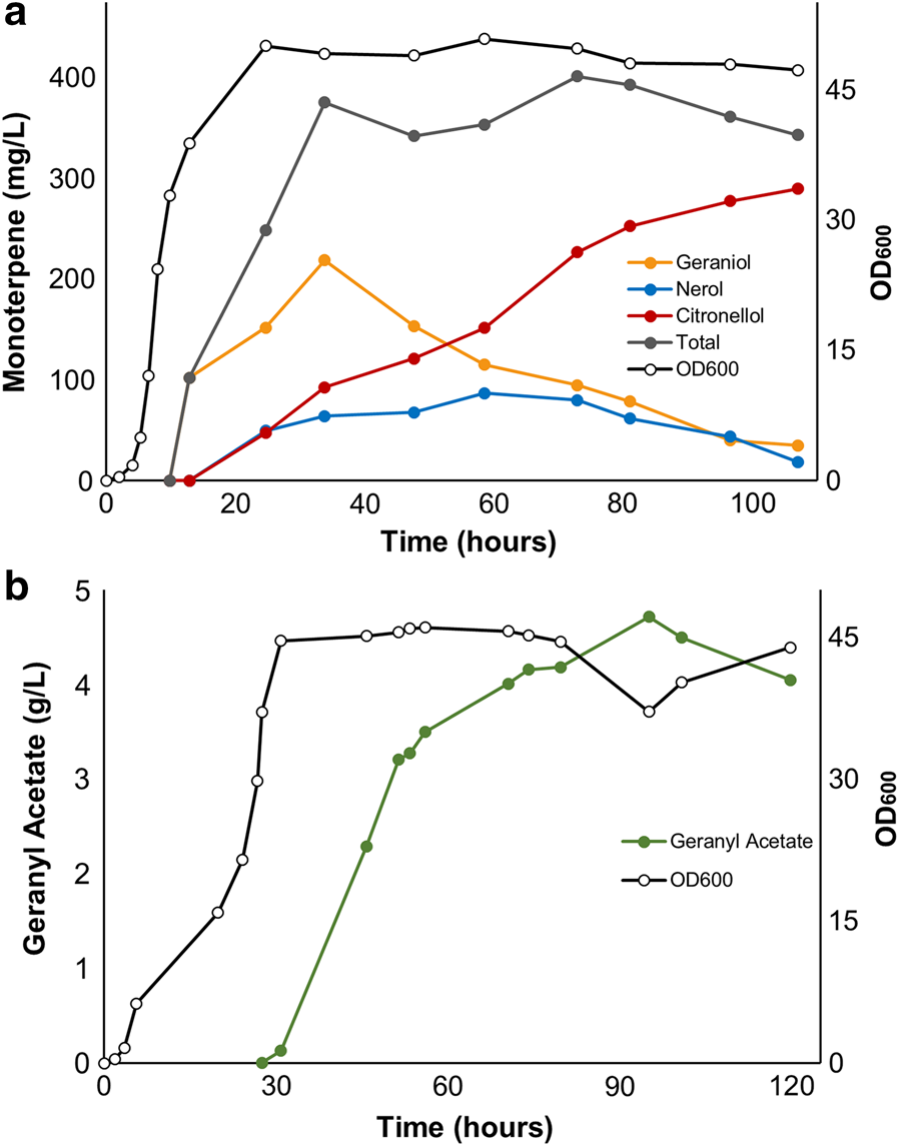
Monoterpene and monoterpene ester production in *E. coli* under fed-batch fermentation using a two-phase system. **a** Accumulation of geraniol (purple), nerol (blue), citronellol (red) and total monoterpene (grey) in *E. coli* strain DLG. **b** Accumulation of geranyl acetate (green) in *E. coli* strain DLGA1. Both cultures were induced at an OD_600_ of ~ 20 with either 50 µM IPTG (**a**) or 125 µM IPTG (**b**), followed by the addition of a 10% dodecane top layer. Culture (**b**) was further supplemented with 20 mM acetic acid. Product titres are based on the aqueous volume of the cultures

**Fig. 5:**
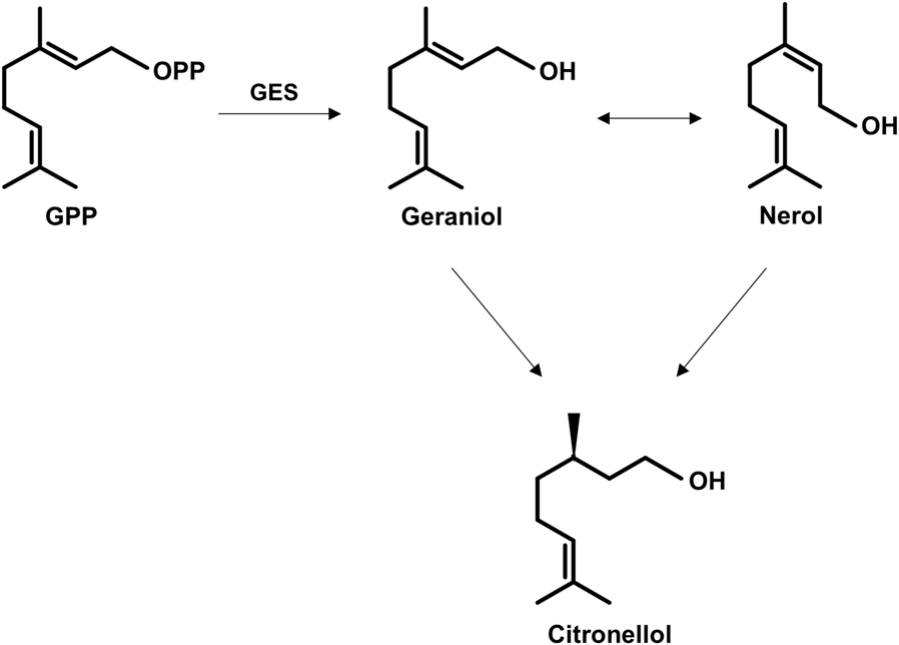
Proposed pathway for the conversion of geraniol to the similar monoterpenoids, nerol and citronellol, by endogenous *E. coli* enzymes. The GES enzyme from *O. basilicum* has exclusive substrate specificity for converting GPP to geraniol. In *E. coli* culture expressing the GES enzyme, the observed accumulation of both nerol and citronellol over time is most likely the result of endogenous enzymes catalysing the isomerization of geraniol to nerol, as well as the reduction of both species to citronellol

To scale up geraniol and geranyl acetate production, fed batch fermentations of strains DLG and DLGA1 were performed in 1.5 L bioreactors. Both strains were cultured in an aqueous-organic biphasic system with a modified TB media (MTB) in the aqueous phase maintained at pH 6.8 by the automatic addition of 5 M KOH. Cultures were grown at 30 °C, and culture OD_600_ and product formation were tracked over the course of 115 h for both strains. With strain DLG, geraniol production peaked at 34 h at 220 mg/L (based on the aqueous volume), after which point geraniol accumulation decreased while citronellol and nerol production increased (Fig. 4a). The reason for the peak in concentration of geraniol at 34 h is not obvious as there was no overt cellular toxicity; possibly, this is the same effect that restricted geraniol production in shake-flask culture. Loss of geraniol from the reactor due to evaporation was discounted as it was found that in the presence of a second organic phase, evaporation was negligible (Additional file 1: Fig. S4). Analysis of total monoterpenoid production suggests that after the peak in geraniol concentration at 34 h, the total monoterpenoid titre in culture remained relatively stable, with only the proportion of each constituent altering over time. This indicates that citronellol and nerol are not by-products of geraniol biosynthesis, but rather arise from the biotransformation of geraniol by endogenous *E. coli* enzymes. To confirm this, we incubated wild-type C43 (DE3) *E. coli* in the presence of either 0.5 mM geraniol or nerol for 6 h and then evaluated the terpene profiles of each culture by GC–MS (Additional file 1: Fig. S3). Following incubation of these cultures with either geraniol or nerol, all three monoterpenoids, geraniol, nerol, and citronellol were detected, supporting the scheme shown in Fig. 5. When wild-type C43 (DE3) *E. coli* was incubated in the presence of 0.5 mM citronellol, no biotransformation to other terpenoids was observed (data not shown), indicating that this is an end-product of geraniol metabolism in *E coli*, as previously observed from studies with *S. cerevisiae* [39]. Possible enzymes responsible for the reduction, isomerization and dehydrogenation of geraniol in *E. coli, S. cerevisiae*, and other organisms have been identified [8, 22, 40]. Over the course of the batch culture of strain DLG, citronellol became the dominant terpene product, accumulating to 290 mg/L after 112 h with minimal concentrations of residual geraniol and nerol remaining (Fig. 4a). Thus, GES expression in *E. coli* could also be considered as a platform for citronellol production, particularly if the endogenous enzymes converting geraniol stepwise to citronellol were over-expressed.

As previously observed in shake flask fermentation, strain DLGA1 produced exclusively geranyl acetate in a two-phase bioreactor operated under fed-batch conditions using MTB media. The culture was fed 20 mM acetic acid directly post induction, in order to increase product titre, as shown in Fig. 3c. Geranyl acetate titre peaked at 95 h at a concentration of 4.8 g/L (based on the aqueous volume) (Fig. 4b), and this could probably be improved by further acetate addition (Fig. 3c). This final titre is 17 times and 13 times the maximum molar concentration of geraniol and citronellol accumulated, respectively, by strain DLG grown under similar conditions.

## Discussion

We have provided proof of concept that the deliberate conversion of the monoterpenoid geraniol to its acetyl ester is an effective way to increase the production of geraniol from engineered whole cell systems, avoiding toxicity, further metabolism of the geraniol, and possible feedback effects on flux through the MEV pathway. Given that the toxicity of monoterpenoids to microbial cells is not limited to geraniol, this approach should be generic, assuming a good AAT can be found for the conversion. This is consistent with the observations of Liu et al. [8] and Tashiro et al. [26], where total geraniol production (both free and esterified) was increased by the fortuitous acetylation of geraniol by a promiscuous chloramphenicol acetyltransferase (CAT) enzyme. However, in both these studies not all geraniol was acetylated, and further metabolism of the geraniol was still observed, suggesting that esterification was occurring more slowly. Further, the lower final titres achieved in both these studies compared to the present work suggests that with incomplete acetylation, the accumulation of geraniol and its monoterpenoid derivatives may result in a product limiting burden on the system, either through negative feedback inhibition and/or toxicity.

Recovery of the terpenoid from the terpenoid ester in the organic phase would require fractional distillation to remove the dodecane, followed by ester hydrolysis to isolate geraniol. Although hydrolysis of geranyl acetate can be readily carried out either in an aqueous or organic environment [41, 42], so the fractional distillation may not be essential. In contrast, isolation of geraniol from a mixture of geraniol, nerol and citronellol via fractional distillation—as would be the case from strain DLG in Fig. 4a—is nearly impossible due to the similar boiling point and vapour pressures of these compounds, and so requires more expensive separation methods [43, 44].

Without any genomic modification of the host strain, the deliberate production of geranyl acetate far exceeds previous titres of geraniol obtained, measured on a molar basis. However, there is still room for improvement. 4.8 g/L geranyl acetate is close to 25 mM, which is similar to the concentration of supplemented acetate. The next step should be to move to a fed-batch mode of acetate supplementation, as was done for glucose (Fig. 4). With no restrictions in flux to geranyl acetate there is no obvious reason why titres in the organic phase could not be increased up to the levels where aqueous phase concentrations become toxic. As this appears to be in the g/L range and the partition between organic and aqueous layers is approx. 24:1, titres of 25–50 g/L should be feasible. However, even that toxicity concentration is questionable as it is clear that *E coli* possesses an acetylesterase (Aes) capable of slowly hydrolysing geranyl acetate to the more toxic geraniol [8], the impact of which would be more obvious at high aqueous geranyl acetate concentrations. Deletion of Aes activity would be a useful target as it would potentially minimize both any geranyl acetate loss due to hydrolysis and any cellular toxicity attributed to geraniol accumulation.

## Conclusion

The commercial production of monoterpene alcohols, such as geraniol, from recombinant microbial hosts, is hampered through the amphiphilic nature of these compounds and their inherent toxicity, which is partly the result of metabolism in the production host. The strategy of deliberate stoichiometric esterification provides an elegant solution to problems of further metabolism, toxicity and product recovery. Using this approach, we have been able to produce 4.8 g/L (aqueous volume) of geranyl acetate as the sole product from glucose in fed batch culture. This represents nearly an order of magnitude of improvement on monoterpenoid production strategies that do not employ esterification [15, 22, 24]. As geranyl acetate is valuable in its own right, this strategy provides a potentially economic route to both products.

This ‘detoxification via esterification’ strategy should be generic and could be applied for the improved production of other commercially valuable monoterpenoids, such as nerol, terpineol, linalool, fenchol, and perillyl alcohol. Further metabolic engineering efforts to improve acetyl-CoA and cofactor availability, and the characterization of additional AAT enzymes will aid in developing this strategy as an economically feasible microbial production platform for monoterpenoids.

## Additional file

**Additional file 1.** Additional methods, figures and tables.

## Data Availability

The datasets used and/or analysed during the current study are available from the corresponding author on reasonable request.
