# Supplementary material for: Esterification of geraniol as a strategy for increasing product titre and specificity in engineered Escherichia coli
Source: Microb Cell Fact. 2019 Jun 8;18:105. doi: 10.1186/s12934-019-1130-0 (PMC6556219; doi:10.1186/s12934-019-1130-0)
Supplement: Supplementary file 1 — Additional file 1. Additional methods, figures and tables. [file 12934_2019_1130_MOESM1_ESM.docx]

ATGAGCTGTGCACGTATTACCGTTACCCTGCCGTATCGTAGCGCAAAAACCAGCATTCAGCGTGGTATTACCC

ATTATCCGGCACTGATTCGTCCGCGTTTTAGCGCATGTACACCGCTGGCAAGCGCAATGCCGCTGAGCAGCAC

ACCGCTGATTAATGGTGATAATTCACAGCGTAAAAACACCCGTCAGCATATGGAAGAAAGCAGCAGCAAACG

TCGTGAATATCTGCTGGAAGAAACCACCCGTAAACTGCAGCGTAATGATACCGAAAGCGTTGAAAAACTGAA ACTGATCGATAACATTCAGCAGCTGGGTATCGGTTATTACTTTGAAGATGCAATTAATGCCGTTCTGCGTAGCC

CGTTTAGCACCGGTGAAGAGGACCTGTTTACCGCAGCACTGCGTTTTCGTCTGCTGCGTCATAATGGTATTGAA

ATTAGTCCGGAAATCTTTCTGAAATTCAAAGACGAACGCGGTAAATTCGATGAAAGCGATACCCTGGGTCTGC

TGAGCCTGTATGAAGCAAGCAATCTGGGTGTTGCGGGTGAAGAAATTCTGGAAGAAGCAATGGAATTTGCAG

AAGCACGTCTGCGTCGTAGCCTGAGCGAACCGGCAGCACCGCTGCATGGTGAAGTTGCACAGGCACTGGATG TTCCGCGTCATCTGCGTATGGCACGTCTGGAAGCCCGTCGTTTTATTGAACAGTATGGTAAACAGAGCGATCA

TGATGGTGACCTGCTGGAACTGGCAATTCTGGATTATAACCAGGTTCAGGCACAGCATCAGAGCGAACTGACC

GAAATTATTCGTTGGTGGAAAGAACTGGGTCTGGTTGATAAACTGAGCTTTGGTCGTGATCGTCCGCTGGAAT

GTTTTCTGTGGACCGTTGGTCTGCTGCCGGAACCGAAATATAGCAGCGTTCGTATTGAACTGGCCAAAGCAAT

TAGCATTCTGCTGGTGATTGATGACATCTTTGATACCTATGGCGAAATGGATGATCTGATTCTGTTTACAGATG CCATTCGTCGTTGGGATCTGGAAGCTATGGAAGGTCTGCCGGAATATATGAAAATTTGTTATATGGCCCTGTAT AACACCACCAATGAGGTTTGTTATAAAGTGCTGCGTGATACCGGTCGTATTGTTCTGCTGAATCTGAAAAGCA CCTGGATCGATATGATTGAAGGTTTTATGGAAGAGGCCAAATGGTTTAATGGTGGTAGCGCACCGAAACTGGA

AGAATATATTGAGAATGGTGTTAGCACCGCAGGCGCATATATGGCATTTGCACATATCTTTTTTCTGATTGGTG

AAGGTGTGACCCATCAGAATAGCCAGCTGTTTACCCAGAAACCGTATCCGAAAGTTTTTAGTGCAGCAGGTCG

TATTCTGCGTCTGTGGGATGACCTGGGCACCGCAAAAGAAGAACAAGAACGCGGAGATCTGGCAAGCTGTGT TCAGCTGTTCATGAAAGAAAAAAGCCTGACCGAAGAAGAAGCCCGTAGCCGTATCCTGGAAGAGATTAAAGG

TCTGTGGCGTGATCTGAATGGTGAACTGGTGTATAACAAAAATCTGCCGCTGTCCATTATCAAAGTTGCACTG

AATATGGCACGCGCAAGCCAGGTTGTGTATAAACATGATCAGGATACCTATTTCAGCAGCGTGGATAATTATG TTGATGCCCTGTTTTTTACCCAGTAA

**Figure S1.** Full-length sequence of the *O. basilicum* GES gene, codon optimized to the genome of *E. coli*.


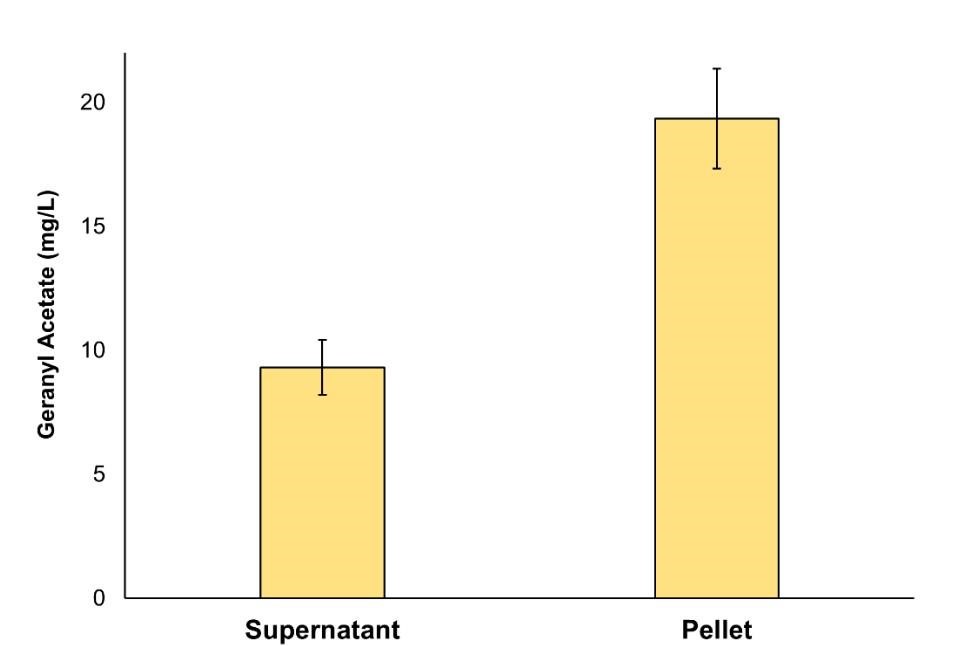


**Figure S2.** Geranyl acetate accumulated in the supernatant and cell pellet of *E. coli* strain DLGA. Cultures were exogenously fed acetic acid and geraniol after induction and grown in a solventfree system for 18 hours at 20°C before analysis. Data are the mean ± standard deviation from three biological replicates.

**Table S1**

*In vitro* activity of the GES enzyme from *O. basilicum* converting GPP to geraniol in the presence of varying concentrations of geraniol. Kinetic parameters were determined using the malachite green assay in the presence of increasing geraniol concentrations. Data are the mean ± standard error from three replicates.

**Geraniol (µM)**

**0**  **100**  **200**  **400**

| ***V*_max_ (µmol/min/mg)** | 1.7 (+ 0.2) | 1.6 (+ 0.2) | 1.5 (+ 0.1) |  | 1.6 (+ 0.1) |
| --- | --- | --- | --- | --- | --- |
| ***K*_m_ (µM)** | 121 (+ 16) | 96 (+ 10) | 98 (+ 12) |  | 98 (+ 14) |
| ***k*cat (s-1)** | 1.8 (+ 0.2) | 1.7 (+ 0.2) | 1.6 (+ 0.1) |  | 1.7 (+ 0.1) |


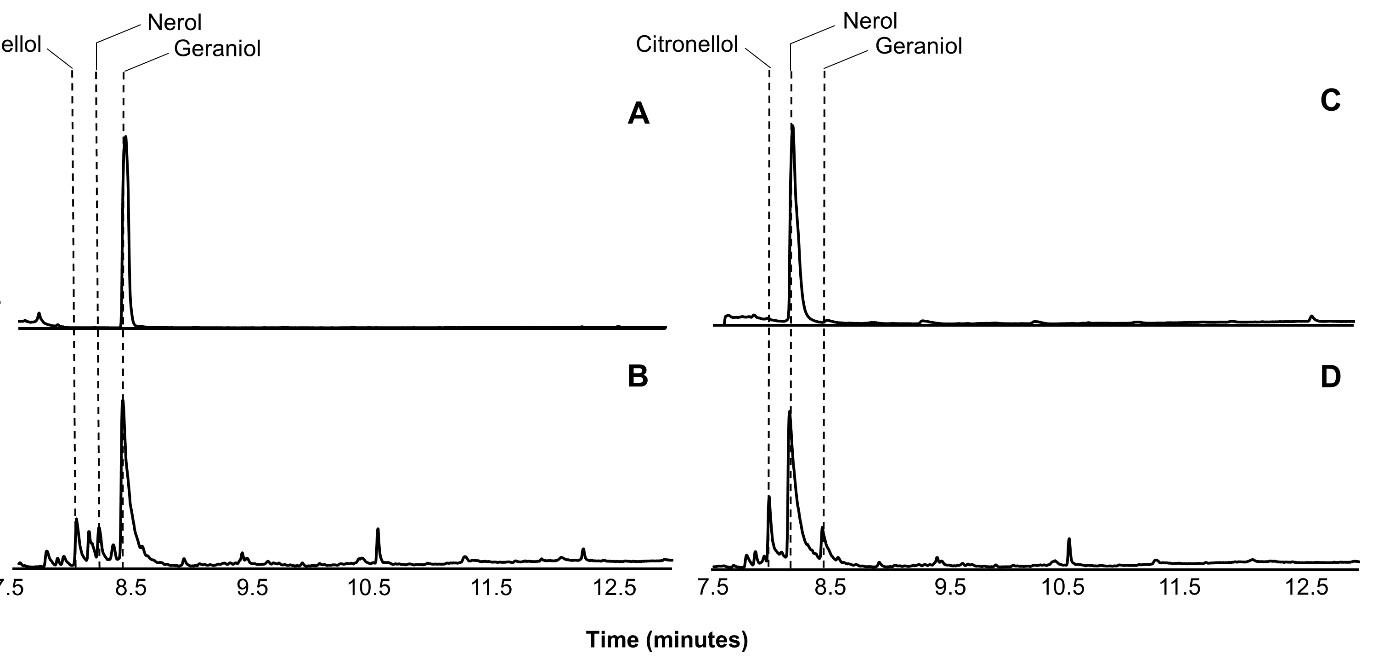


**Figure S3.** Gas chromatograms showing the monoterpenes formed in *E. coli* C43 (DE3) after incubation in the presence of either geraniol or nerol. Panels **A** and **C** show internal standards of geraniol and nerol, respectively. Panel **B** and **D** show the monoterpenes present in the cell pellet of wild type *E. coli* C43 (DE3) incubated in the presence of either 0.5 mM geraniol or 0.5 mM nerol respectively, for 6 h. The peaks corresponding to citronellol, nerol and geraniol are indicated.


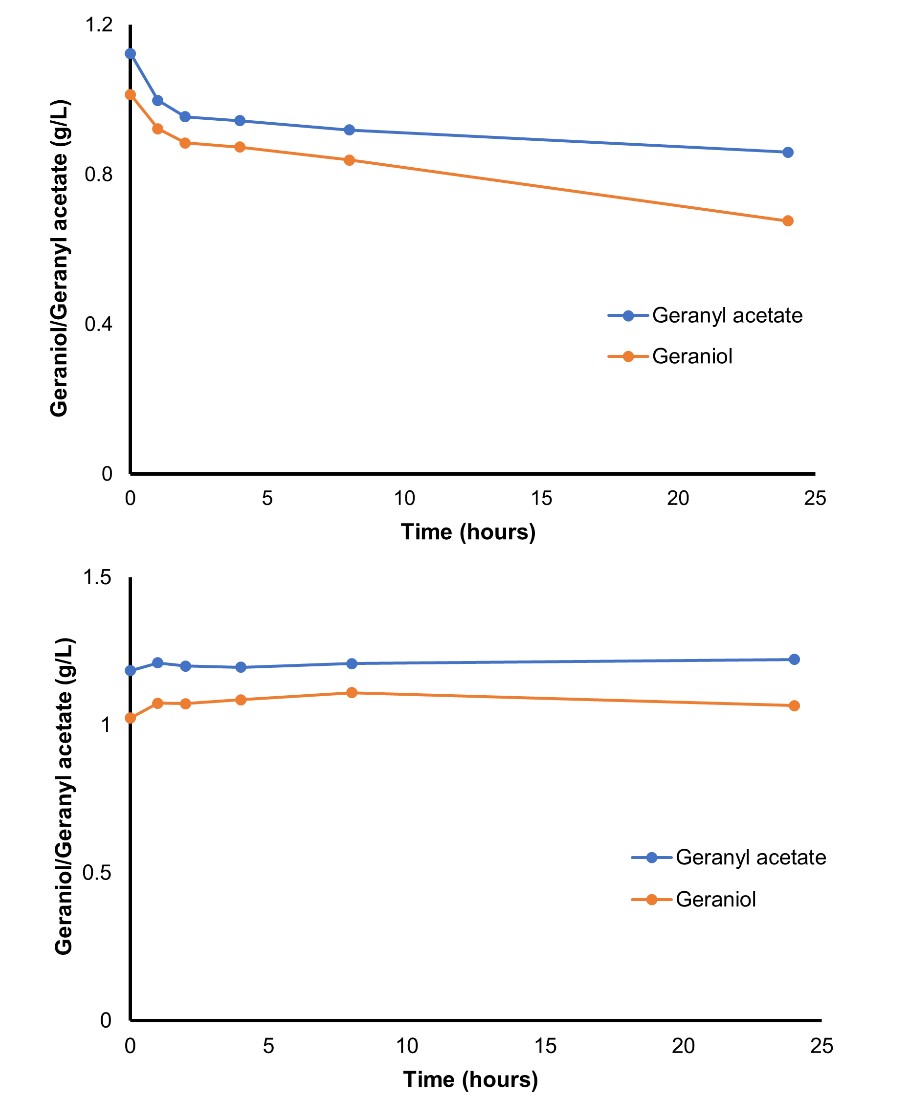


**Figure S4.** Time course of geraniol and geranyl acetate evaporation. Top panel shows the amount of geraniol and geranyl acetate present in a solvent-free system over 24 hours from an initial addition of ~1 g/L for each. Bottom panel shows the amount of geraniol and geranyl acetate present in a two-phase system – with total geraniol and geranyl acetate representing a combination of the proportions in the aqueous and organic phases.


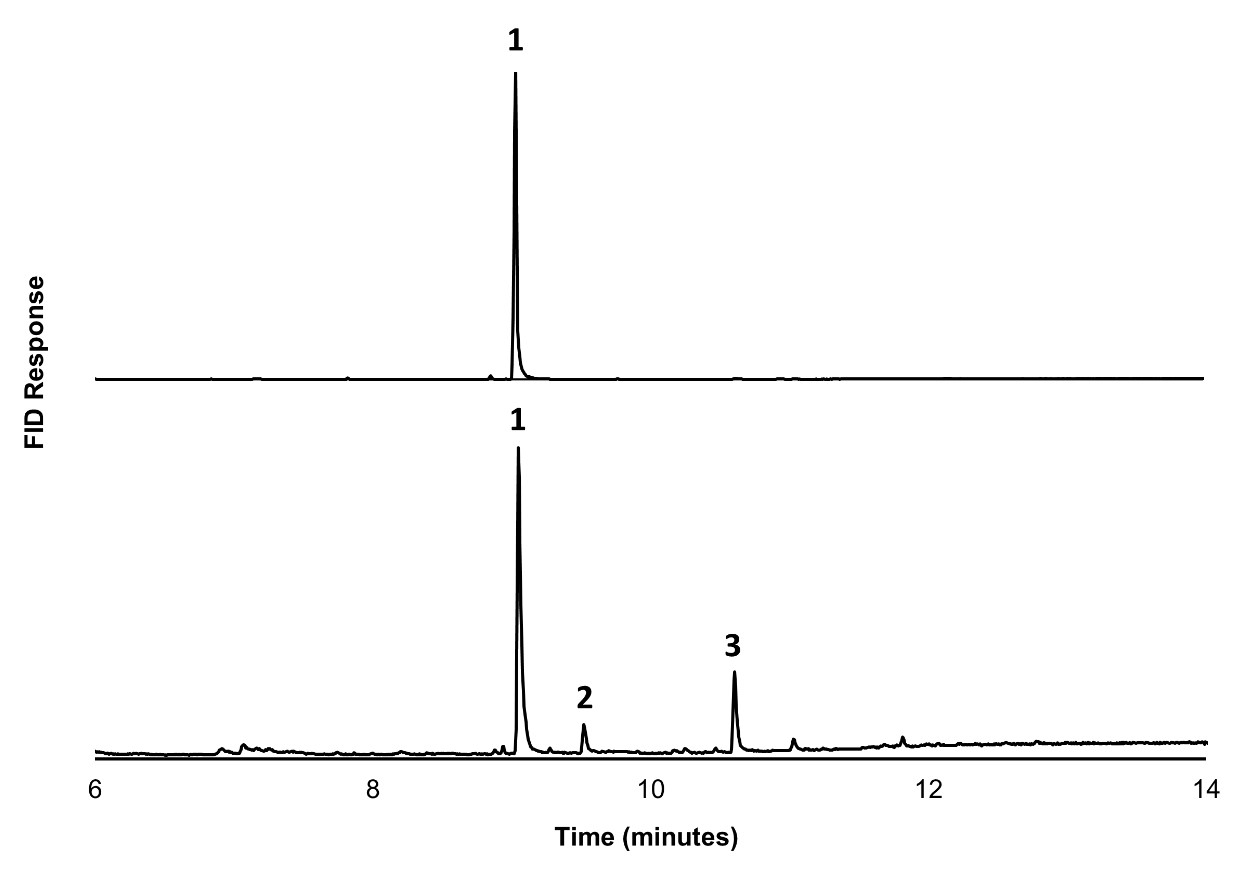


**Figure S5.** Gas chromatograms showing the endogenous hydrolysis of geranyl acetate to geraniol in *E. coli* C43 (DE3) after geranyl acetate feeding. Top panel shows an internal standard of geranyl acetate. Bottom panel shows the terpene products present in the culture supernatant of wild type *E. coli* C43 (DE3) incubated in the presence of 0.25% geranyl acetate for 6 hours. 1 = geranyl acetate; 2 = geraniol; 3 = 2-octen-1-ol, 3,7, dimethyl, -isobutyrate (Z).

**
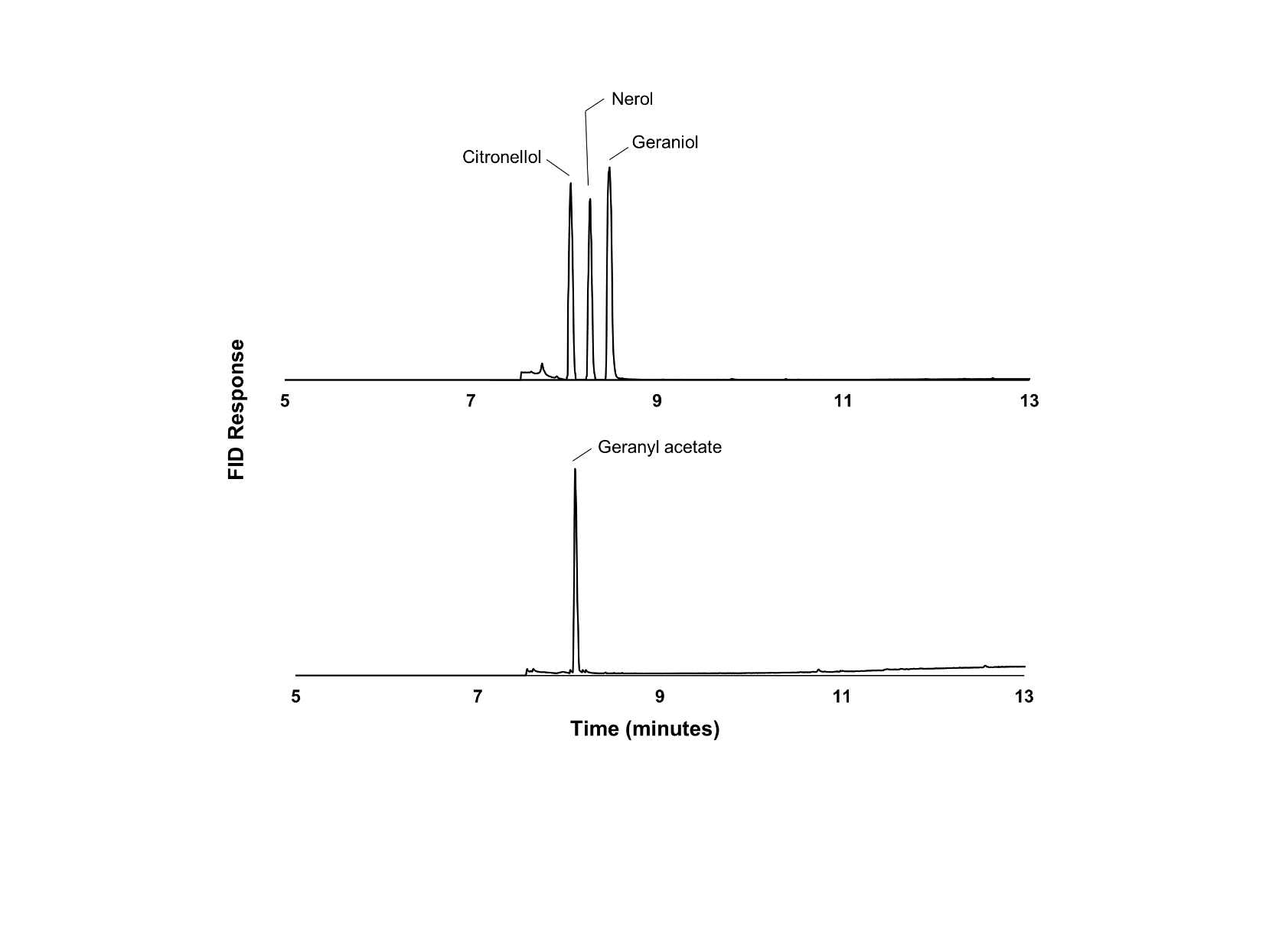
**

**Figure S6.** Gas chromatograms showing the retention times for citronellol, nerol, and geraniol standards (top panel), and geranyl acetate (bottom panel).

**Table S2**

Amount of geraniol, nerol, citronellol and geranyl acetate produced by *E. coli* strains DLG and DLGA1 after 24 hours in the experiment described in Figure 3B.

| **Strain** | **Geraniol (mg/L)** | **Nerol (mg/L)** | **Citronellol (mg/L)** | **Geranyl acetate (mg/L)** |
| --- | --- | --- | --- | --- |
| **DLG** | 34.9 | 8.6 | 14.8 | -- |
| **DLGA1** | -- | -- | -- | 347 |

**Table S3**

Amount of geraniol, nerol, citronellol and total monoterpene produced by *E. coli* strain DLG during the fed-batch time course shown in Figure 5A.

| **Time (mins)** | **Geraniol (mg/L)** | **Nerol (mg/L)** | **Citronellol (mg/L)** | **Total monoterpene (mg/L)** |
| --- | --- | --- | --- | --- |
| 12.9 | 102.2 | 0 | 0 | 0 |
| 24.7 | 152.1 | 49.5 | 47.3 | 248.8 |
| 33.8 | 218.9 | 63.9 | 92.7 | 375.6 |
| 47.6 | 153.5 | 67.7 | 121.2 | 342.4 |
| 58.6 | 115.1 | 86.9 | 151.7 | 353.7 |
| 72.9 | 94.8 | 80 | 226.9 | 401.6 |
| 81.1 | 78.7 | 61.7 | 252.8 | 393.2 |
| 96.6 | 39.9 | 43.7 | 277.8 | 361.3 |
| 107 | 35 | 18.5 | 289.9 | 343.4 |

**Table S4**

Amount of geranyl acetate produced by *E. coli* strain DLGA1 during the fed-batch time course shown in Figure 5B.

| **Time (min)** | **Geranyl Acetate (g/L)** |
| --- | --- |
| **27.5** | 0 |
| **45.8** | 2.3 |
| **51.9** | 3.3 |
| **56** | 3.6 |
| **74** | 4.3 |
| **95** | 4.8 |
| **100.8** | 4.6 |
| **119** | 4.1 |

**Methods**

# Expression of the R. hybrida AAT in E. coli

*E. coli* harbouring plasmid pET28a::AAT was inoculated into LB broth supplemented with kanamycin (50 µg/ml) and grown overnight at 37°C. Cultures were then inoculated at 1% into 25 ml of TB + 2% glucose medium with kanamycin (50 µg/ml) and grown at 37°C until an OD_600_ of 1 was reached.

Cultures were then induced with 400 µM IPTG and moved to 20°C and incubated in a rotary shaker (250 rpm) for 2 hours before being fed 10 mM acetic acid and 0.5 mM geraniol. Cultures were then further incubated for 18 hours at 20°C and 250 rpm. The lower temperature was used to minimize terpene volatilization. After 18 hours the OD_600_ was measured and the culture was centrifuged at 13,000 rpm at 4°C to separate the supernatant and pellet fractions. The supernatant was directly extracted into hexane for analysis by gas-chromatography mass-spectometry (GC-MS). The pellet was resuspended in 0.9% NaCl, sonicated at 12 microns on ice until clear, and then extracted into hexane for analysis by GC-MS.

# Analysis of endogenous isomerization and reduction of geraniol and nerol in E. coli

*E. coli* strain C34(DE3) was inoculated into LB broth and grown overnight at 37°C. The following day, cultures were inoculated at 1% into 5 ml of TB + 2% glucose medium and grown at 37°C in a rotary shaker (250rpm) until an OD_600_ of 4 was reached. Cultures were then fed either 0.5 mM geraniol or 0.5 mM Nerol and further incubated at 37°C for 6 h before being centrifuged at 13,000 rpm to separate the culture supernatant and pellet. The pellet was resuspended in 0.9% NaCl, sonicated at 12 microns on ice until clear, and then extracted into hexane for analysis of monoterpene products by GC-MS.

# GES enzyme purification and in vitro assays

*E. coli* strain BL21(DE3) (New England Biolabs) containing the plasmid pET28a::AAT was grown at 37°C in LB broth supplemented with kanamycin (50 µg/ml) overnight. The culture was then inoculated to 1% in 1 L of Terrific Broth (TB) with kanamycin (50 µg/ml) and grown at 37°C and 250 rpm until an OD_600_ of 1.0 was reached. At this point gene expression was induced with the addition of 50 µM IPTG and cultures were further incubated for another 16 hours at 30°C. The culture was then centrifuged at 4000 rpm for 20 minutes at 4°C and the cell pellet was resuspended in Buffer A (20 mM Tris pH 8.0, 300 mM NaCl, 10 mM imidazole) and sonicated on ice at 12 microns until clear. The soluble lysate fraction was run through a Poly-Prep chromatography column (0.8 x 4 cm, Bio-Rad, Watford, UK) with a pre-equilibrated metal affinity resin charged with cobalt (Clontech, Takara Bio Europe, SaintGermainen-Laye, France). Following this, the His(6x)-tagged AAT was then eluted into a 2 ml mixture of Buffer A and Buffer B (20 mM Tris pH 8.0, 300 mM NaCl, 1 M imidazole) containing a final imidazole concentration of 500 mM. Protein concentration was determined using a Bradford assay.

The malachite green assay was performed in 96-well flat bottom plates in a total volume of 50 µl according to Vardakou *et al.,* (2014). The assay mix contained malachite green assay buffer (25 mM MES, 20 mM CAPS, 50 mM Tris, 2.5 mU of inorganic pyrophosphatase (*Saccharomyces cerevisiae,* Sigma-Aldrich, Dorset, UK), 5 mM MgCl_2_) pH 7.5, 0.008 µM purified GES enzyme, geranyl pyrophosphate (GPP) ranging in concentration from 10-400 µM, and a fixed concentration of geraniol (either 0, 100, 200, or 400 µM). Reactions were set up on ice and incubated at 30°C for 5 minutes. The reaction was halted by the addition of 12 mL of malachite green development solution and incubated a further 15 minutes at 30°C prior to reading at 623 nm on a BioTek microplate reader. Malachite green development solution was prepared by mixing 10 ml of malachite green dye stock solution (300 ml of

18 M H_2_SO_4_ mixed with 1.5 L of water and 2.2 g of malachite green powder) with 2.5 ml of 7.5% ammonium molybdate, and 0.2 ml 11% Tween 20. Standard curves of monophosphate (Pi) and pyrophosphate (PPi) ranging from 0.39-50 µM were used for product quantification. Kinetic parameters were calculating using Sigma Plot graphing software (Systat software Inc.).

# Geraniol and geranyl acetate volatility

A 1.5 L bioreactor (BIOSTAT B, Sartorius, Germany) containing 1.5 L of 89 mM potassium phosphate buffer pH 6.8 +/- 10% dodecane top layer, was inoculated with 1 g/L of geraniol and 1 g/L geranyl acetate. Temperature was maintained at 30 °C and dissolved oxygen was maintained at 20% saturation through combined control of air flow and stirrer speed (maintained between 0.5-1 L/min and 300-600 rpm, respectively). Over a time course of 24 hours, samples of the aqueous and organic phases were taken and prepared for analysis by GC-MS for quantification of the amount of geraniol and geranyl acetate present in each phase.

# Endogenous hydrolysis of geranyl acetate by E. coli

*E. coli* strain C43 (DE3) was inoculated into LB broth and grown overnight at 37°C. Cultures were then inoculated to 1% (v/v) into 10 ml TB + 20 g/L glucose medium and grown at 37°C until an OD_600_ of 4.0 was reached, at which point the culture was supplemented with 0.25% (v/v) geranyl acetate and further incubated at 37°C in a rotary shaker for 6 hours. Culture supernatant was sampled and extracted into hexane for analysis of endogenous geranyl acetate hydrolysis by gas chromatography-mass spectrometry

(GC-MS).
